# Weight-control compensatory behaviors patterns and correlates: a scoping review

**DOI:** 10.3389/fpsyg.2024.1383662

**Published:** 2024-10-14

**Authors:** Trista Yue Yuan, Narges Bouzari, Andy Bains, Tamara R. Cohen, Lisa Kakinami

**Affiliations:** ^1^Faculty of Land and Food Systems, University of British Columbia, Vancouver, BC, Canada; ^2^BC Children’s Research Institute, BC Children’s Hospital, Vancouver, BC, Canada; ^3^Department of Mathematics and Statistics, Concordia University, Montreal, QC, Canada; ^4^School of Health, Concordia University, Montreal, QC, Canada

**Keywords:** weight-control, compensatory health behaviors, compulsive exercise, drunkorexia, disordered eating, weight management

## Abstract

**Objective:**

Weight-control compensatory behaviors appear to be a commonly utilized strategy for health management. Individuals engaging in such behaviors believe that the negative consequences from unhealthy behaviors will be neutralized by the positive consequences of healthy behaviors. Existing research has not reached a consensus on whether such behaviors are beneficial to health. This review aims to (1) summarize the patterns of weight-control compensatory health behaviors in different populations, (2) highlight correlates, predictors, and consequences of compensatory health behaviors, and (3) identify gaps for future research.

**Method:**

This review identified existing literature using online databases, CINAHL and PubMed. Primary research articles published after 2000 with non-clinical participants of 12 years or older who engaged in compensatory behaviors for weight control purposes were selected. Descriptive statistics were extracted from 35 studies.

**Results:**

Different patterns for weight-control compensatory behaviors emerged between the female and male sexes. Meanwhile, no clear association of such behaviors was found across weight status. Studies reviewed also highlighted three main areas of compensatory behaviors for weight management, namely dietary behaviors, physical activity, and alcohol consumption. Weight-control compensatory behaviors had significant negative correlations with mental health indicators, such as psychosocial functioning, emotional differentiation ability, and body esteem.

**Conclusion:**

Weight-control compensatory behaviors may be a widely used weight management strategy and can be presented in diverse ways. Although believed to be promoting health, such behaviors appear to be associated with poor psychological well-being. This emerging topic warrants more in-depth investigation to establish the direction of causation. Future research may investigate the relationship between weight-control compensatory behaviors and various aspects of health over longer time periods, examine the engagement of multiple weight-control compensatory behaviors, and focus on high-risk populations.

## 1 Introduction

The World Health Organization has defined health as a state of complete physical, mental and social well-being and not merely the absence of disease or infirmity (World Health Organization, 1948). Weight is commonly used as a quantitative indicator of health outcomes. Healthcare systems utilizing a weight-centric framework focus on weight management and promote weight loss as a prevention and treatment to a variety of chronic noncommunicable diseases, such as diabetes, heart diseases, and chronic pain (Mauldin et al., 2022; Tylka et al., 2014). Weight-control compensatory behaviors are “corrective” actions that a person employs to counteract the effects of behaviors believed to cause weight gain (Colleen Stiles-Shields et al., 2012). This behavior stems from the belief that the negative consequences of “unhealthy” behaviors will be neutralized by the positive consequences of “healthy” behaviors (Knäuper and Rabiau, 2004). Individuals following a weight-centric framework may often attempt to improve their health by controlling their weight via lifestyle changes, such as modifying dietary and physical activity habits. A subtype of such compensatory behaviors, termed drunkorexia, happens in response, and/or anticipation of alcohol consumption, where adults modify lifestyle behaviors for alcohol consumption to avoid unintended weight gain (Griffin and Vogt, 2021; Roosen and Mills, 2015).

A recent concept analysis review article analyzed the underlying motivations of holding compensatory health beliefs (Zhao et al., 2021). The authors concluded that individuals trying to manage their weight may use compensatory beliefs as a way to minimize the cognitive dissonance when their behaviors do not align with their weight management intentions (Zhao et al., 2021). However, research also suggests that compensatory health *beliefs* may not equate to compensatory health *behaviors* (Amrein et al., 2017; Forestier et al., 2020; Radtke et al., 2014)_._ Less is known about the relationship between compensatory health behaviors and health.

To date, studies on compensatory behaviors and health are mixed. While some report that those with greater compensatory health behaviors tend to have poorer health (Buchholz and Crowther, 2014; Castañeda et al., 2020; Wammes et al., 2007), others report null findings or positive health effects (Amrein et al., 2017; Sob et al., 2021). This heterogeneity stems from the variety of populations being studied, and a synthesis of these weight-focused compensatory health behaviors is needed. Thus, the overall goal of this scoping review was to focus on investigating who uses compensatory health behaviors and identify the health correlates of using such behaviors. Specifically, this review aims to (1) summarize the patterns of compensatory health behaviors in different populations, (2) highlight correlates, predictors, and consequences of compensatory health behaviors, and (3) identify gaps for future research.

## 2 Method

### 2.1 Search strategy

Electronic searches of two databases [Cumulative Index to Nursing and Allied Health Literature (CINAHL) and PubMed] were conducted between July 2020 and June 2021. All databases were accessed through EBSCOhost. The initial search was conducted through CINAHL in July 2020; in July 2021 and August 2023, the search was updated on CINAHL and expanded to include PubMed. The search strategy included compensatory health behaviors relevant to weight and appearance control by combining compensatory health behavior terms with eating behavior terms and physical activity terms. The search strategy and search terms are provided in Table 1. As this study is an assessment of previously published research, ethical approval is not required.

**Table 1:** Search terms and strategy.

#	Query
S1	(MH “Contingency Management”)
S2	TI (“compensatory behavio*” OR “contingency management”) OR AB (“compensatory behavio*” OR “contingency management”)
S3	S1 or S2
S4	(MH “Eating Behavior+”)
S5	(MH “Diet+”)
S6	TI (“eating behavio*” OR diet* OR fasting OR “food habit*” OR “food preference*” OR “portion size*”) OR AB (“eating behavio*” OR diet* OR fasting OR “food habit*” OR “food preference*” OR “portion size*”)
S7	S4 OR S5 OR S6
S8	(MH “Physical Activity”) OR (MH “Exercise+”)
S9	TI (“physical activit*” or exercis*) OR AB (“physical activit*” or exercis*)
S10	S8 OR S9
S11	TI (“Instrument Construction” OR “Instrument Validation”) OR AB (“Instrument Construction” OR “Instrument Validation”)
S12	(MH “Instrument Construction+”) OR (MH “Instrument Validation”) OR (MH “Instrument Scaling+”)
S13	(MH “Research Measurement+”)
S14	TI (“research measurement*” OR index* OR Indices OR Scale*) OR AB (“research measurement*” OR index* OR Indices OR Scale*)
S15	S11 OR S12 OR S13 OR S14
S16	S3 AND S7 AND S15
S17	S7 OR S10
S18	S3 AND S15 AND S17

S#: Search #; MH: Subject Heading, TI: Title; AB: Abstract.

### 2.2 Eligibility criteria

Although weight control behaviors have been reported for elementary- and middle-school aged children, research suggests that recall in youth below age 12 may not be accurate (Diep et al., 2015). Thus, this review focused on adolescents and adults (i.e., ≥12 years). Other eligibility criteria for potential study inclusion were: (1) examination of compensatory health behaviors focused on weight management and associated factors, and (2) primary research articles published in peer-reviewed journals in the English language after 2000. The year 2000 was selected as the compensatory health belief measure was initially developed in 2004 (Knäuper and Rabiau, 2004) and compensatory health behaviors were unlikely to have been systematically measured too many years prior to 2004. Studies were excluded for the following reasons: (1) sampled from clinical populations, (2) animal studies, (3) experimental studies, and (4) focused on the development and validation of measurements related to compensatory behaviors. The implications of these criteria for our findings are described in the discussion.

### 2.3 Selection process

Two reviewers independently screened the titles and abstracts of identified citations for potential eligibility. When the eligibility could not be determined based on the title and abstract, the articles were then read in their entirety. Disagreements between the two reviewers were resolved by discussion, and when a consensus could not be reached, a third reviewer weighed in on the final decision. Reference lists of eligible studies were examined to further identify potential studies for inclusion. This process was repeated until no additional articles were identified as relevant for inclusion.

### 2.4 Data extraction

Data extracted from each study included: authors, year of publication and country, study design, sample size, participant demographic characteristics, measures used to assess weight management compensatory behaviors, other measures (such as psychosocial correlates), study findings, and study strengths and limitations.

### 2.5 Assessment of study quality

The quality of the studies was assessed in accordance with the Appraisal tool for Cross-Sectional Studies (AXIS) and included items such as the inclusion of study objectives, study design and method, description of analyses, and adequate reporting of results (Downes et al., 2016). A value of ‘yes’ indicated that the item was addressed; the total number of items with a ‘yes’ was calculated for each study. As a ‘yes’ for items 13 and 19 of the AXIS measure indicated poorer quality, these were reverse-coded. Two to three reviewers independently assessed the quality of the articles. Disagreements between the reviewers were resolved by discussion and when a consensus could not be reached, the corresponding author weighed in on the final decision.

## 3 Results

### 3.1 Search results

**Figure 1:**
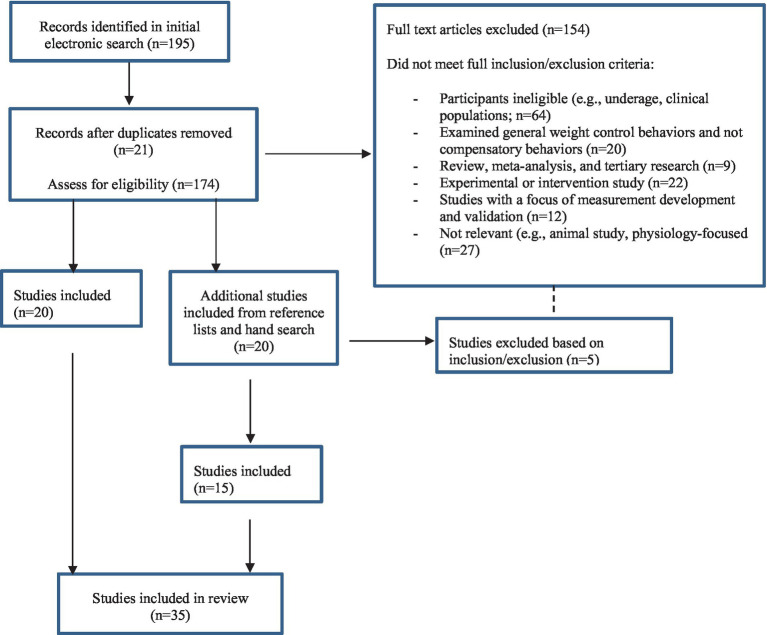
Review strategy decision tree.

The initial search (June 2020) produced 96 records. The updated search conducted in July 2021 produced 11 new records from CINAHL and 65 articles on PubMed, and an additional 15 articles were included from the reference lists of the eligible studies. The second updated search conducted in August 2023 produced 15 records from CINAHL and 8 articles on PubMed. After the removal of duplicates and ineligible articles, 35 publications were included (Figure 1).

#### 3.1.1 Study characteristics

Table 2 presents a summary of the data extracted for each study sample. Twenty-three studies were conducted in the USA (Anderson and Bulik, 2004; Bankoff et al., 2013; Blackstone and Herrmann, 2020; Bryant et al., 2012; Buchholz and Crowther, 2014; Burke et al., 2010; Castañeda et al., 2020; Dunn et al., 2003; Eisenberg et al., 2017; Eisenberg and Fitz, 2014; Eneva et al., 2017; Giles et al., 2009; Gorrell et al., 2019; Hill and Lego, 2019; Holmes et al., 2023; Hoover et al., 2023; Horvath et al., 2020; Hunt and Forbush, 2016; Martin et al., 2016; Michael and Witte, 2021; Peralta and Barr, 2016; Staples and Rancourt, 2022; Williams-Kerver and Crowther, 2020), three in Italy (Laghi et al., 2019; Lupi et al., 2014; Pompili and Laghi, 2018), one in Canada (Roosen and Mills, 2015), one in the UK (Griffin and Vogt, 2021), one in the Netherlands (Wammes et al., 2007), one in Switzerland (Sob et al., 2021), and two in Australia (Knight et al., 2017; Moeck and Thomas, 2021). Three studies included multi-country samples (Choquette et al., 2018; Fuller-Tyszkiewicz et al., 2022; McCabe et al., 2023). One study used random sampling (Giles et al., 2009) while all others used convenience sampling (Anderson and Bulik, 2004; Bankoff et al., 2013; Blackstone and Herrmann, 2020; Bryant et al., 2012; Buchholz and Crowther, 2014; Burke et al., 2010; Castañeda et al., 2020; Choquette et al., 2018; Dunn et al., 2003; Eisenberg et al., 2017; Eisenberg and Fitz, 2014; Eneva et al., 2017; Fuller-Tyszkiewicz et al., 2022; Gorrell et al., 2019; Griffin and Vogt, 2021; Hill and Lego, 2019; Holmes et al., 2023; Hoover et al., 2023; Horvath et al., 2020; Hunt and Forbush, 2016; Knight et al., 2017; Laghi et al., 2019; Lupi et al., 2014; Martin et al., 2016; McCabe et al., 2023; Michael and Witte, 2021; Moeck and Thomas, 2021; Peralta and Barr, 2016; Pompili and Laghi, 2018; Roosen and Mills, 2015; Sob et al., 2021; Staples and Rancourt, 2022; Wammes et al., 2007; Williams-Kerver and Crowther, 2020).

**Table 2:** Studies examining weight-control compensatory behaviors in cross-sectional and longitudinal studies (*N* = 35).

Author(s); year	Research question	Country	*N* (% women); M_age_	Compensatory behavior measure (independent [IV] or dependent [DV] variable)	Statistical analysis	Results (validated measurements for correlates/predictors in square brackets)
Anderson and Bulik (2004)	Assess sex differences on use of compensatory behaviors.	USA	2,621 (57% F, twins), age of sample not provided,	Own measure (DV, separately, and the sum of the following during a binge eating period): vomiting, laxatives, diuretics, diet pills, exercise, fasting	Logistic regression	Compared to men, women were significantly (*p* < 0.05) more likely to report using the following compensatory behaviors: vomiting (OR: 3.37), laxatives (OR: 2.88), diuretic (OR: 4.57), diet pills (OR: 3.34), fasting (OR: 2.02).
Bankoff et al. (2013)	Investigate relationship between childhood abuse experiences and adult relationship dynamics on the use of compensatory behaviors in adulthood.	USA	759 (100% F); 19.2 years	Own measure of at least one of the following (DV): purging/vomiting; use of laxatives or diuretics; use of diet pills	Logistic regression IV: childhood abuse; global psychosocial functioning; relationship anxiety; relationship avoidance; sexual relationship power	†Relationship avoidance [ECR-R], global psychosocial functioning [GSI subscale of BSI], and an interaction between childhood abuse [CTQ-SF] and relationship avoidance were significantly associated with the use of at least one compensatory behavior.
Blackstone and Herrmann (2020)	Determine whether compensatory behaviors are associated with exercise dependence when step goal or caloric output goal from a fitness wearable is not met.	USA	337 (100% F); 19.3 years	Own measure (IV, separate binary variables): (a) eating less the next day; (b) increasing physical activity; (c) delaying going to sleep; (d) exercising more vigorously	Linear regression among those who wear fitness wearables (*n* = 198) Confounders: EPSI	Compensating for not following step goal by: increasing vigorous exercise and restricting food had significantly higher exercise dependence scores (B = 0.383; *p* < 05; B = 0.172; *p* < 0.05, respectively). Compensating for not meeting caloric output was not detected.
Bryant et al. (2012)	Assess sex differences in compensatory behaviors related to alcohol consumption.	USA	274 (81% F), 21.5 years	CEBRACS (DV)	Chi-square tests	Compared to men, women were more likely to eat low-calorie or low-fat food prior to drinking, during drinking, and after drinking to compensate for the calories (all *p* < 0.05). Compared to men, women were more likely to report eating less than usual before drinking, or skipping meals after drinking to compensate for the calories (both *p* < 0.05).
Buchholz and Crowther (2014)	Investigate relationships between women who use exercise as a compensatory behavior in alcohol and exercise behavior.	USA	206 (100% F), 19.5 years	Own measure (IV, 2 items): if at least one of these two items: ‘do you exercise [before/after] a drinking episode in order to reserve these calories for your intended alcohol consumption’	ANOVA	Compared to women who did not use exercise as a compensatory behavior for their drinking, those who did significantly differed in: (a) motivations for drinking [DMQ] (coping, conformity, enhancement); (b) motivations for exercise compensatory behavior [FES] (appearance/weight concerns, health and enjoyment); (c) and greater dietary restraint, and appearance dissatisfaction (all *p* < 0.05).
Burke et al. (2010)	Determine whether calorie restriction on days a student planned to drink was associated with number of days alcohol was consumed.	USA	692 (68% F), 18.6 years	Own measure (IV, 3 items, Cronbach’s alpha: 0.86): item details not provided but was focused on calorie restriction on days a person intended to drink	Chi-square tests	Number of days alcohol was consumed was significantly different between current drinkers and binge drinkers who restricted calories when they were drinking compared to those who did not restrict calories.
Castañeda et al. (2020)	Determine which CEBRACS subscale is associated with binge drinking frequency.	USA	1,149 (67.2% F), college freshmen, age not provided	CEBRACS (IV, as separate subscales in a single model)	Structural equation path analysis Confounder: sex	CEBRACS subscales of dietary restraint and bulimia were both significantly associated with binge drinking frequency (B = 0.21, B = −0.92, respectively; both *p* < 0.05).
Choquette et al. (2018)	Comparison of alcohol related compensatory behaviors between the US and France.	USA	502 (73% F American), 21 years; 365 (68% F French), 21.4 years	CEBRACS (DV, total score)	Linear regression IV: (model 1): nationality; AUDIT; nationality*AUDIT; (Model 2): nationality; drive for thinness; nationality*drive for thinness	†Greater alcohol use [AUDIT-C], nationality, and their interaction term were all significantly associated with higher CEBRACS scores. Greater drive for thinness [EDI-DT] and its interaction with nationality were both significantly associated with higher CEBRACS scores.
Dunn et al. (2003)	Determine whether readiness for change of general eating behaviors, binge eating, or compensatory behaviors are differentially associated with compensatory behaviors.	USA	175 (65% F), 18.5 years	EDDS compensatory behaviors (DV; mean of four items)	Linear regression IV: readiness for change of: (a) general eating behaviors; (b) binge eating; (c) compensatory behaviors	General eating behaviors readiness for change, binge eating readiness for change, and compensatory behaviors readiness for change were all marginally associated (*p* = 0.05) with compensatory behaviors.
Eisenberg and Fitz (2014)	Assess sex differences in compensatory behaviors related to alcohol consumption and their motivations for compensatory behaviors	USA	63 (64% F), 19.6 years	Own measure: (1) frequency (DV): number of days the participant ‘restricted food or calories before drinking alcohol’; (2) motivation (DV): how often they restricted food, calories, or carbohydrates on days they planned to drink in order to avoid weight gain	(1) ANCOVA Confounder: number of alcoholic drinks (2) Linear regression Confounder: number of alcohol drinks; weight control motivations; their interaction	(1) Compared to men, women had significantly more compensatory behavior days related to alcohol consumption. Weight control mediated the relationship between sex and number of compensatory behavior days related to alcohol consumption. (2) Among women, stronger weight control motivations and heavier alcohol consumption were significantly associated with compensatory behaviors related to alcohol consumption.
Eisenberg et al. (2017)	Assess sex differences in compensatory behaviors related to alcohol consumption.	USA	410 (64% F), 19.8 years	FRAC (IV, 1 item): how many days in the past month they restricted food, calories, or carbs before drinking alcohol	Linear regression Confounders: sexual objectification; sex; and their interaction term	†Women, and the interaction between sex and sexual objectification [ISOS] were associated with FRAC (*p* < 0.05).
Eneva et al. (2017)	Assess sex differences in compensatory behaviors related to reward and punishment sensitivity	USA	1,022 (64% F), 21 years	EDDS (DV)	Linear regression Confounders: sex and interaction with SPSRQ	†Women were more likely to use compensatory behaviors than men. A significant interaction between sex and reward sensitivity [SPSRQ] suggested that women were more likely to use compensatory behaviors at high levels of reward sensitivity.
Fuller-Tyszkiewicz et al. (2022)	Test and refine an integrated cross-sectional theoretical model of disordered eating and appearance change behaviors across 8 countries.	Australia Belgium Canada China Italy Japan Spain USA	6,272 (67% F), 21.5 years	IV: DSM-5 and PHQ-2 DV: DMS, DLS, EDE-Q	Hybrid exploratory/confirmatory approach exploratory structural equation modelling Multi-group path analysis Chi-square, CFI, and RMSEA to evaluate model fit across groups	Greater engagement in compensatory behaviors was more common for individuals who identified as heterosexual, reported a previous eating disorder, felt pressure from peers to lose weight, overvalued their appearance, more strongly internalized the muscular ideal, engaged in physical activity, emotional eating, muscle gaining and weight lose behaviors more regularly, and who reported higher sleep quality.
Giles et al. (2009)	Determine association between compensatory behavior related to alcohol consumption and likelihood of intoxication.	USA	2,873 (58% F), 20.6 years	Own measure (IV, 5 pt. Likert scale): ‘how often they restricted food, fat, or calories on days they planned to drink alcohol’	Logistic regression Confounders: age; race; in a sorority/fraternity; whether the student was an athlete; whether the student lived on campus; within school clustering Modeled separately by sex	Among men, students with compensatory behaviors related to alcohol consumption were 99% more likely to get drunk in a typical week. Among women, students with compensatory behaviors related to alcohol consumption were 2.37 times more likely to get drunk in a typical week.
Gorrell et al. (2019)	Investigate sex differences in compensatory behaviors related to alcohol consumption and eating disorder pathology.	USA	530 (48% F), 18.96 years [men]; 19.48 years [female]	CEBRACS (IV, continuous)	Linear regression Confounders: alcohol frequency; alcohol quantity; BMI; Sex interaction	†Dietary restraint/exercise CEBRACS subscale was significantly associated with EDDS score.
Griffin and Vogt (2021)	Determine whether body esteem or sensation seeking were associated with compensatory behaviors related to alcohol consumption.	UK	95 (86% F), 21.4 years	CEBRACS (DV, total continuous score)	Linear regression IV: BSSS; BESAA	Greater sensation seeking [BSSS] and appearance esteem [BESAA] subscale were significantly associated with greater CEBRACS (both *p* < 0.05).
Hill and Lego (2019)	Determine whether seeking out novel experiences, and body self-esteem are associated with compensatory behaviors related to alcohol consumption.	USA	488 (70% F), 19.6 years	CEBRACS (DV)	Linear regression Predictors: sensation-seeking [BSSS], body esteem [BESAA], and their interaction terms with one another	Greater interest in seeking novel and varied experiences, (B = 0.21) and lower self-esteem scores (B = −0.16 for BESAA-appearance, and B = −0.17 for BESAA weight, respectively) had significantly higher CEBRACS scores (all *p* < 0.05).
Holmes et al. (2023)	Examine the associations among intimate partner violence (IPV), posttraumatic stress disorder symptoms (PTSD), and disordered eating (DE) among women intimate partner violence survivors residing in shelter.	USA	212 (100% F), 34.64 years	CAS_R_-SF, DSM-5 (PCL-5 and EDDS)	Mediation analyses of the direct and indirect effects of IPV on DE through PTSD symptoms Repeated for DE outcomes: weight/shape concerns, binge symptoms, and compensatory behaviors.	Higher levels of IPV [CAS_R_-SF] were associated with greater PTSD [DSM-5 (PCL-5)] and DE [EDDS]. IPV was significantly associated with weight/shape concerns and binge symptoms (*p* < 0.05). IPV had a direct impact on compensatory behaviors, but no indirect influence through severity of PTSD symptoms was observed.
Hoover et al. (2023)	Investigate if the perceived vulnerability to disease (PVD) subscales are associated with fear of fat (FOF), cognitive restraint and compensatory behaviors.	USA	247 (46.7% F), 36.8 years	DV: PVD (perceived infectability and perceived germ aversion) IV: Goldfarb FOF, DEBQ, EDDS	Correlational analyses Mediation analyses to see if FOF mediated the association between PVD subscales, CR, and CB	Perceived infectability [PVD] was associated with cognitive restraint and compensatory behaviors and FOF [FOF] partially mediated these associations. Perceived germ aversion was not related to compensatory behaviors but has significant association with cognitive restraint and FOF did not significantly mediate this association. No differences in sex was observed.
Horvath et al. (2020)	Examine associations between emotion dysregulation with compensatory behaviors related to alcohol consumption.	USA	417 (52% F), 19.3 years	CEBRACS (subscales as separate DV models)	Binomial regression IV: DERS; EDE-Q; AUDIT; BMI; sex; sex*DERS	Emotion dysregulation [DERS] was not significantly associated with CEBRAS total or subscales.
Hunt and Forbush (2016)	Determine whether disordered eating or drinking was associated with compensatory behaviors related to alcohol consumption.	USA	579 (53% F); 19.4 years	Own measure (DV, sum of 5-pt Likert scale; Cronbach’s alpha: 0.82): (a) skipped meal, (b) ate less before drinking, (c) restricted eating prior to drinking to increase the effects of alcohol, (d) strenuous exercise, (e) drank excessively in order to vomit food	Linear regression IV: AUDIT; EPSI Tested in full sample and separately by sex	EPSI subscales of cognitive restraint, purging, and excessive exercise were significantly associated with compensatory behaviors (B = 0.21, B = 0.32, B = 0.07, respectively; all *p* < 0.05). Results were more pronounced among women
Knight et al. (2017)	Identify correlates of compensatory behaviors related to alcohol consumption.	Australia	136 (100% F), 21.3 years	CEBRACS (DV)	Linear regression Confounders: age; ethnicity; binge drinking; EDDS	Binge drinking, and EDDS scores were significantly associated with CEBRACS (both *p* < 0.05).
Laghi et al. (2019)	Determine whether eating behavior psychological measures are associated with compensatory behaviors related to alcohol consumption.	Italy	849 (39% F), 17.9 years	CEBRACS (DV)	Linear regression Confounders: age; BMI	Greater mood instability and impulsivity scores [emotional dysregulation of EDI-3] (B = 0.17) and greater tendency to restrain oneself and be disciplined [asceticism of EDI-3] (B = 0.10) were associated with greater CEBRACS (both *p* < 0.05).
Lupi et al. (2014)	Explore the relationship between eating habits and alcohol consumption.	Italy	1,311 (53% F), 22.05 years	No details of measure provided.	Chi-square tests	No statistically significant differences in eating behaviors between those who consumed alcohol and those who did not, or between binge drinkers and non-binge drinkers.
Martin et al. (2016)	Determine the correlates of compensatory behaviors related to alcohol consumption.	USA	683 (57% F), 20.4 years	Own measure (DV; “How often do you cut back on eating before drinking alcohol to avoid gaining weight”; 5-pt Likert scale, binary: 0 vs. 1+)	Logistic regression (among drinkers only, *n* = 489) Confounders: sex; race; whether the student was an athlete; hazardous drinking level (AUDIT) score	Each unit higher AUDIT score, and men, were 40% more likely, and 2.5 times more likely (respectively) to report cutting back on eating before drinking alcohol in order to avoid gaining weight (both *p* < 0.05).
McCabe et al. (2023)	Identify biopsychosocial factors that predict higher BMI and disordered eating across 7 countries	Europe North America Australia Japan	846 (78.13% F), 22.78 years	DV: EDE-Q, DMS, DLS	Full information maximum likelihood to deal with missing data Multivariate linear regression (6 models, one for each outcome) with gender as a fixed predictor	There was a significant variation in the BMI and disordered eating predictors among the regions and outcomes that were studied. Between western and eastern cultures, there seem to be significant variations in the factors linked to eating-related behaviors and weight status.
Michael and Witte (2021)	Examine the association between trauma symptoms and alcohol-related disordered eating	USA	478 (74.9% F), 18.96 years	IV: PDS, CAGE, EDDS DV: CEBRACS [alcohol effects subscale and alcohol related weight control: composite score of the other three subscales ( α =0.92)]	Bivariate correlational analyses Regression Controlling for biological sex, BMI, factors related to eating disorders and alcohol use disorder.	PTSD symptoms predicted compensatory behaviors (*p* < 0.01), even after adjusting for factors of eating and alcohol disorder.
Moeck and Thomas (2021)	Establish food and alcohol disturbance (FAD) rates and examine the gender differences in adult sample.	Australia	253 (49% F), 3,871 years	IV: EAT-26, AUDIT, IPAQ-SF DV: CEBRACS, ICB-WGA	Multivariate linear regression Hierarchical linear regression *t*-test to compare hazardous and non-hazardous alcoholic drinkers	FAD rates were particularly higher among high-risk drinkers and excessive exercisers. No gender difference was observed but men were found to be more likely to engage in FAD/weight-control.
Peralta and Barr (2016)	Determine whether agreement with gender norms was associated with compensatory behaviors related to alcohol consumption.	USA	651 (59.95% F), 19.8 years	CEBRACS (DV)	Ordinal logistic regression Confounders: sex; parental education; race; age; whether the participant was a sexual minority; whether the participant lived off-campus; depression score, smoking status, drug use, heavy episodic drinking + 3 measures assessing gender norms in separate models	Higher scores of acceptance of gender norms [BSRI] and masculine ideals [BSRI] were associated with higher CEBRACS scores, but associations were attenuated when controlling for depression, smoking, and drug use.
Pompili and Laghi (2018)	Determine the relationship between eating behaviors and compensatory behaviors related to alcohol consumption.	Italy	823 (56% F), 17.9 years	CEBRACS (DV)	Linear regression Confounders: age; BMI; drive for thinness; binge drinking; number of drunkenness episodes in the past month; EDI	Greater likelihood to use laxatives to control weight (B = 0.42), drive for thinness (B = 0.15), binge drinking (B = 0.50) and number of drunkenness episodes in the past month (B = 2.35) were all significantly associated with greater CEBRACS scores (all *p* < 0.05).
Roosen and Mills (2015)	Investigate motivations and correlates of compensatory behaviors related to alcohol consumption.	Canada	Study 1: 3409 (70% F), 19.6 years Study 2: 226 (100% F), 19.7 years	Own measure (if at least one of any of these items prior to drinking alcohol): (a) I eat less food, (b) I skip a meal, (c) I skip more than one meal, (d) I eat no food	Chi-square tests Tested for sex differences	Compared to those who reported eating more food prior to drinking alcohol, those who said they eat less or skip meals were significantly more likely to be motivated to avoid weight gain (Study 1), have higher restraint scores [RS], higher EDEQ scores, higher anxiety (BAI), and depression scores [BDI-II] (Study 2, all *p* < 0.05). Men were more likely to report eating more food prior to drinking alcohol, and women were more likely to report eating less food (Study 1, *p* < 0.05).
Sob et al. (2021)	Whether compensatory behaviors at baseline predicted diet quality, BMI, or physical activity 2-years later.	Switzerland	Baseline: 5238 (49% F), 57 years; Longitudinal: 2638 (51% F), 59 years	Own measure (IV, mean of 7-pt Likert scale; Cronbach’s alpha: 0.82): (a) go to the gym, (b) eat less calories, (c) smaller food portions, (d) choose food with fewer calories, (e) more fruit and vegetables, (f) skip breakfast	Linear regression at follow-up based on baseline values Confounders: age; sex + diet quality, BMI, or physical activity for their respective follow-up values	Greater compensatory behaviors at baseline significantly predicted higher diet quality and physical activity (B = 0.05, B = 0.04, respectively, both *p* < 0.01). Greater compensatory behaviors at baseline not predictive of BMI at follow-up.
Staples and Rancourt (2022)	Test the interaction of thinness and negative affect expectancies, along with its association with disordered eating behavior	USA	401 (55.5% F), 21 years	IV: EEI, TREI, EPSI DV: EDDS (binge eating and compensatory behaviors)	Correlation analyses Negative binomial regression Exploratory indirect effect models	Thinness/restriction expectancies and negative affect expectancies were significantly positively correlated with compensatory behaviors. Compared to males, females reported greater negative affect and thinness/restriction expectancies and more compensatory behaviors.
Wammes et al. (2007)	Describe timing, and types of compensatory behaviors used based on weight status and socioeconomic status	The Nether-lands	857 (52% F); 27 years	Own measure (DV, compensatory behavior by eating less; compensatory behavior by being more physically active): timing: (day before overeating, same day, day after, within a few days); meal occasion: (breakfast, lunch, dinner)	Descriptive (*n* = 276); no statistical results with the compensatory behaviors data provided	Higher proportions compensated by eating less (42%) rather than being more physically active (23.9%). Most compensated the same day as overeating (14.1%) or the day after (13.4%) rather than before (2.5%). Compensating with more physical activity occurred the day after overeating (14.9%) rather than the same day (13.8%).
Williams-Kerver and Crowther (2020)	Determine whether better differentiation between negative emotions, and positive emotions were associated with compensatory behaviors.	USA	118 (100% F), 19.4 years	EDDS (DV: sum of items 15–18)	Linear regression IV: appearance schemas; negative affect intensity; positive affect intensity; negative emotion differentiation; positive emotion differentiation; and their interactions terms with appearance schemas	†Poorer ability to differentiate between negative emotions, stronger appearance schemas, and their interaction term were all significantly associated with higher compensatory behaviors.

AUDIT: Alcohol Use Disorders Identification Test; AUDIT-C: Alcohol Use Disorders Identification Test of Consumption; BAI: Beck Anxiety Inventory; BDI-II: Beck Depression Inventory-II; BESAA: Body Esteem Scale for Adolescents and Adults Scale; BSI: Brief Symptom Inventory; BSRI: Ben Sex Role Inventory; BSSS: Brief Sensation Seeking Scale; CAGE: Cut, Annoyed, Guilty, and Eye (alcohol-use disorder questionnaire); CAS_R_-SF: Composite Abuse Scale-Short Form; CMNI: Conformity to masculine norms inventory; CTQ-SF: Short form of Childhood Trauma Questionnaire; DEBQ: Dutch Eating Behavior Questionnaire; DERS: Difficulties in Emotion Regulation Scale; DMQ: Motivation: Drinking Motives Questionnaire; DMS: Drive for Muscularity Scale; DLS: Drive for Leanness Scale; DSM-5: The Diagnostic and Statistical Manual of Mental Disorders, fifth edition; EAT-26: Eating Attitudes Test; ECR-R: Experiences in Close Relationships-Revised Questionnaire Scale; EDDS: Eating Disorder Diagnostic Scale; EDE-Q: Eating Disorder Examination-Questionnaire; EDI-3: Eating Disorders Inventory-3; EDI-DT: Eating Disorder Inventory – Drive for Thinness Subscale; EDS-21: Exercise Dependence Scale-21; EEI: Eating Expectancy Inventory; EPSI: The Eating Pathology Symptom Inventory; FES: Function of Exercise Scale; FOF: Fear of Fat scale; FRAC: Food-Restricted Alcohol consumption; GSI: Global Severity Index; ICB-WGA: Inappropriate Compensatory Behaviors to Avoid Weight Gain from Consuming Alcohol; IPAQ-SF: International Physical Activity Questionnaire Short Form; ISOS: Interpersonal Sexual Objectification Scale; PAQ: Personal attributes questionnaire; PDS: Posttraumatic Diagnostic Scale; PHQ-2: Parent Health Questionnaire (second edition); PCL-5: PTSD Checklist; PVD: Perceived Vulnerability to Disease Questionnaire; RRS: Restraint Scale – Revised; SPSRQ: Sensitivity to Punishment/Sensitivity to Reward Questionnaire; TREI: Thinness and Restricting Expectancy Inventory. †Significant interaction term in the regression model.

Twenty-three studies included university students (Bankoff et al., 2013; Blackstone and Herrmann, 2020; Bryant et al., 2012; Buchholz and Crowther, 2014; Burke et al., 2010; Castañeda et al., 2020; Choquette et al., 2018; Dunn et al., 2003; Eisenberg et al., 2017; Eisenberg and Fitz, 2014; Eneva et al., 2017; Giles et al., 2009; Gorrell et al., 2019; Hill and Lego, 2019; Horvath et al., 2020; Hunt and Forbush, 2016; Knight et al., 2017; Martin et al., 2016; Michael and Witte, 2021; Peralta and Barr, 2016; Roosen and Mills, 2015; Staples and Rancourt, 2022; Williams-Kerver and Crowther, 2020), six studies recruited community adult samples (age ranging from 18–100 years old) (Holmes et al., 2023; Hoover et al., 2023; Lupi et al., 2014; Moeck and Thomas, 2021; Sob et al., 2021; Wammes et al., 2007), two studies targeted adolescents (Sob et al., 2021; Wammes et al., 2007), one study employed twin pair samples and did not specify age (Anderson and Bulik, 2004), three studies had a mixed-age sample (students, non-students, and former students) (Fuller-Tyszkiewicz et al., 2022; Griffin and Vogt, 2021; McCabe et al., 2023). Two studies were longitudinal (McCabe et al., 2023; Sob et al., 2021) and the other 33 articles were cross-sectional (Anderson and Bulik, 2004; Bankoff et al., 2013; Blackstone and Herrmann, 2020; Bryant et al., 2012; Buchholz and Crowther, 2014; Burke et al., 2010; Castañeda et al., 2020; Choquette et al., 2018; Dunn et al., 2003; Eisenberg et al., 2017; Eisenberg and Fitz, 2014; Eneva et al., 2017; Fuller-Tyszkiewicz et al., 2022; Giles et al., 2009; Gorrell et al., 2019; Griffin and Vogt, 2021; Hill and Lego, 2019; Holmes et al., 2023; Hoover et al., 2023; Horvath et al., 2020; Hunt and Forbush, 2016; Knight et al., 2017; Laghi et al., 2019; Lupi et al., 2014; Martin et al., 2016; Michael and Witte, 2021; Moeck and Thomas, 2021; Peralta and Barr, 2016; Pompili and Laghi, 2018; Roosen and Mills, 2015; Staples and Rancourt, 2022; Wammes et al., 2007; Williams-Kerver and Crowther, 2020). The specific findings on patterns, correlates, predictors, and consequences of weight-control compensatory behaviors, including drunkorexia, are broadly summarized (Table 2).

#### 3.1.2 Quality and risk of bias assessments

Table 3 presents the results of the quality assessments. None of the studies provided a power calculation or sample size justification. Most of the studies (*n* = 21) did not provide enough information to determine whether there was a potential non-response bias (e.g., no response rate, and/or no measures addressing missing data). The lack of the aforementioned information lowered the quality and rigor of the eligible studies. Weight-control compensatory behaviors were measured by either validated eating disorder measures [e.g., Eating Disorder Examination Questionnaire (EDE-Q)] (Mond et al., 2004). Eating Disorder Diagnostic Scale (EDDS) (Fairburn and Beglin, 1994; Stice et al., 2000) or by questionnaires that are created by study authors to answer a specific question (Anderson and Bulik, 2004; Bankoff et al., 2013; Blackstone and Herrmann, 2020; Buchholz and Crowther, 2014; Burke et al., 2010; Giles et al., 2009; Hunt and Forbush, 2016; Martin et al., 2016; Roosen and Mills, 2015; Sob et al., 2021; Wammes et al., 2007). One exception to this are the measurements related to alcohol-related weight-control behaviors, which are often measured using the Compensatory Eating Behaviors Related to Alcohol Consumption Scale (CEBRACS) (Rahal et al., 2012). Twelve studies used measures that were not validated and did not present reliability and validity psychometric properties. The majority of studies declared no conflicts of interest, 10 studies did not report such information, and two studies stated potential sources of conflict of interest. Publication bias was suspected to be minimal as studies reporting null findings were included in this review.

**Table 3:** Quality assessment of included studies using the quality of cross-sectional studies (AXIS) tool.

	Quality assessment†																				
	1	2	3	4	5	6	7	8	9	10	11	12	13R	14	15	16	17	18	19R	20	Total score
Anderson and Bulik (2004)	Y	Y	N	Y	N	N	N	Y	N	Y	Y	N	N	N	Y	Y	Y	Y	DK	DK	9/20
Bankoff et al. (2013)	Y	Y	N	Y	Y	N	N	Y	N	Y	Y	Y	DK	N	N	Y	Y	Y	Y	Y	13/20
Blackstone and Herrmann (2020)	Y	Y	N	Y	N	N	N	Y	N	Y	Y	Y	DK	N	N	Y	N	Y	Y	Y	11/20
Bryant et al. (2012)	Y	Y	N	Y	N	N	N	Y	N	Y	N	Y	DK	N	Y	N	Y	Y	Y	Y	11/20
Buchholz and Crowther (2014)	Y	Y	N	Y	N	N	Y	Y	Y	Y	Y	Y	N	Y	Y	Y	Y	Y	DK	Y	15/20
Burke et al. (2010)	Y	Y	N	Y	N	N	N	Y	N	Y	N	Y	N	N	N	Y	N	Y	DK	DK	8/20
Castañeda et al. (2020)	Y	Y	N	Y	N	N	Y	Y	Y	Y	Y	Y	N	N	Y	Y	Y	Y	DK	Y	14/20
Choquette et al. (2018)	Y	Y	N	Y	N	N	N	Y	Y	Y	Y	Y	DK	N	Y	Y	N	Y	DK	Y	12/20
Dunn et al. (2003)	Y	Y	N	Y	N	N	N	Y	N	Y	Y	Y	DK	N	Y	Y	Y	Y	DK	Y	12/20
Eisenberg and Fitz (2014)	Y	Y	N	Y	N	N	Y	Y	Y	Y	Y	Y	DK	N	N	Y	Y	Y	Y	Y	14/20
Eisenberg et al. (2017)	Y	Y	N	Y	N	N	N	Y	N	Y	Y	Y	DK	N	N	Y	Y	Y	Y	Y	12/20
Eneva et al. (2017)	N	Y	N	Y	N	N	N	Y	Y	Y	Y	Y	DK	N	N	Y	Y	Y	N	Y	11/20
Fuller-Tyszkiewicz et al. (2022)	Y	Y	N	Y	N	N	N	Y	Y	Y	Y	Y	DK	N	Y	Y	Y	Y	Y	Y	14/20
Giles et al. (2009)	Y	Y	N	Y	Y	Y	N	Y	N	Y	Y	Y	N	N	Y	Y	Y	Y	N	Y	14/20
Gorrell et al. (2019)	Y	Y	N	Y	N	N	N	Y	Y	Y	Y	Y	DK	N	N	Y	Y	Y	Y	Y	13/20
Griffin and Vogt (2021)	Y	Y	N	Y	N	N	Y	Y	Y	Y	Y	Y	DK	N	N	Y	Y	Y	Y	Y	14/20
Hill and Lego (2019)	Y	Y	N	Y	N	N	Y	Y	Y	Y	Y	Y	DK	N	Y	Y	Y	Y	Y	Y	15/20
Holmes et al. (2023)	Y	Y	N	Y	Y	N	N	Y	Y	Y	Y	Y	Y	N	Y	Y	Y	Y	Y	Y	16/20
Hoover et al. (2023)	Y	Y	N	Y	N	N	N	Y	Y	Y	Y	Y	DK	N	N	Y	Y	Y	Y	Y	13/20
Horvath et al. (2020)	Y	Y	N	Y	N	N	Y	Y	Y	Y	Y	Y	Y	N	Y	Y	Y	Y	DK	Y	15/20
Hunt and Forbush (2016)	Y	Y	N	Y	N	N	N	Y	N	Y	Y	Y	DK	N	Y	Y	Y	Y	Y	Y	13/20
Knight et al. (2017)	Y	Y	N	Y	N	N	Y	Y	Y	Y	Y	Y	DK	Y	Y	Y	Y	Y	Y	Y	16/20
Laghi et al. (2019)	Y	Y	N	Y	N	N	N	Y	Y	Y	Y	Y	DK	N	N	Y	Y	Y	Y	Y	13/20
Lupi et al. (2014)	Y	Y	N	Y	N	N	N	Y	N	Y	N	Y	DK	N	N	N	Y	N	DK	Y	8/20
Martin et al. (2016)	Y	Y	N	Y	N	N	Y	Y	Y	Y	Y	Y	N	N	Y	Y	Y	Y	DK	Y	14/20
McCabe et al. (2023)	Y	Y	N	Y	N	N	Y	Y	Y	Y	Y	Y	Y	Y	N	Y	Y	Y	Y	Y	16/20
Michael and Witte (2021)	Y	Y	N	Y	N	N	N	Y	Y	Y	Y	Y	N	N	Y	Y	Y	Y	Y	Y	14/20
Moeck and Thomas (2021)	Y	Y	Y	Y	N	N	N	Y	Y	Y	N	Y	DK	N	Y	N	Y	Y	Y	Y	13/20
Peralta and Barr (2016)	Y	Y	N	Y	N	N	Y	Y	Y	Y	Y	Y	N	Y	Y	Y	Y	Y	Y	Y	16/20
Pompili and Laghi (2018)	Y	Y	N	Y	N	N	N	Y	Y	Y	Y	Y	DK	N	N	Y	Y	Y	Y	Y	13/20
Roosen and Mills (2015)	Y	Y	N	Y	N	N	Y	Y	N	Y	Y	Y	DK	N	N	Y	Y	Y	Y	Y	13/20
Sob et al. (2021)	Y	Y	N	Y	Y	Y	Y	Y	N	Y	Y	Y	N	N	Y	Y	Y	Y	Y	Y	16/20
Staples and Rancourt (2022)	Y	Y	N	Y	N	N	Y	Y	Y	Y	Y	Y	N	N	Y	Y	Y	Y	Y	Y	15/20
Wammes et al. (2007)	Y	Y	N	Y	N	N	N	Y	N	Y	Y	Y	N	N	N	Y	Y	Y	Y	DK	11/20
Williams-Kerver and Crowther (2020)	Y	Y	N	Y	N	N	Y	Y	N	Y	Y	Y	DK	Y	Y	Y	Y	Y	DK	Y	14/20

†In accordance with the AXIS tool. R: ITEM was reverse-coded. 1. Introduction: were the aims/objectives clear; 2. Methods: was the study design appropriate; 3. Methods: was the sample size justified; 4. Methods: Was the target/reference population clearly defined; 5. Methods: was the sample frame taken from an appropriate population base; 6. Methods: was the selection process likely to select participants that were representative of the target population under investigation; 7. Methods: were the risk factor and outcome variables measured appropriate; 8. Were the risk factor and outcome variables measured appropriate to the aims of the study; 9. Methods: were the risk factor and outcome variables measured correctly using instruments that had been piloted or published previously; 10. Methods: is it clear what was used to determine statistical significance; 11. Methods: were the methods sufficiently described; 12. Results: were the basic data adequately described; 13R. Results: were concerns of non-response bias minimal; 14. Results: if appropriate, was information about non-responders described; 15 Results: were the results internally consistent; 16. Results: were the results presented for all the analyses described; 17. Discussion: were the authors’ discussions and conclusions justified by the results; 18. Discussion: were the limitations of the study discussed; 19R. Other: were concerns of conflicts of interest (funding or otherwise) minimal; 20. Other: was ethical approval or consent attained.

### 3.2 Patterns of weight-control compensatory behaviors based on individual characteristics

#### 3.2.1 Biological sex

The studies investigating the interaction of compensatory behaviors with sex (*n* = 7) in general reported that while both males and females engaged in weight-focused compensatory behaviors, females tended to employ compensatory behaviors more frequently than males (Bryant et al., 2012; Burke et al., 2010; Eisenberg and Fitz, 2014; Gorrell et al., 2019; Staples and Rancourt, 2022; Wammes et al., 2007) although not always (Fuller-Tyszkiewicz et al., 2022). However, unique patterns emerged across sexes: males were more likely to modify their physical activity levels while females were more likely to change their eating patterns as a compensatory strategy (Anderson and Bulik, 2004; Wammes et al., 2007). In response to binge eating or overeating, rates of compensatory behavior were similar between males and females in some studies (Eneva et al., 2017), but not in others (Anderson and Bulik, 2004; Wammes et al., 2007).

Sex differences were identified with alcohol consumption frequency, but different patterns emerged between sexes in relation to drinking frequency and drunkorexia. While there was a consensus across study findings that males tended to drink more alcohol than females, there are conflicting findings on whether that accounts for sex differences between the frequency of drunkorexia engagement. The majority of these studies (*n* = 7) found no significant differences in frequency of drunkorexia engagement between males and females (Bryant et al., 2012; Castañeda et al., 2020; Gorrell et al., 2019; Griffin and Vogt, 2021; Horvath et al., 2020; Moeck and Thomas, 2021; Peralta and Barr, 2016) while four found a higher frequency in females (Eisenberg et al., 2017; Eisenberg and Fitz, 2014; Martin et al., 2016; Roosen and Mills, 2015). One study found for females, greater alcohol consumption was associated with a higher frequency of drunkorexia (adjusted for weight status). This relationship was not found with light alcohol consumption, showing that those most at risk of drunkorexia are females who both consume a lot of alcohol and have a substantial weight control motive, regardless of their weight status (Burke et al., 2010). In addition, one study found gender orientation (independent of biological sex) was associated with drunkorexia such that masculine-oriented (i.e., presenting oneself more as masculine) individuals were at more significant risk for drunkorexia (Peralta and Barr, 2016).

Sex differences with the behavioral categories and correlates of drunkorexia were observed. Males tended to exercise more after the alcohol consumption, while females tended to restrict calories before their alcohol consumption, reflecting that there may be temporality differences when it comes to different sexes engaging in drunkorexia (Bryant et al., 2012; Giles et al., 2009; Gorrell et al., 2019). While these differences were minor because males and females participate in both, there were unique correlates associated with females’ drunkorexia behaviors. For instance, alcohol-related purging (i.e., vomiting, using laxatives/diuretics) only increased eating disorder symptomatology among females (Gorrell et al., 2019). Furthermore, interpersonal sexual objectification significantly interacted with drunkorexia for females but not males. In other words, the more females reported interpersonal sexual objectification (e.g., viewing and treating one as objects for sexual desire), the more they restricted food for alcohol consumption (Eisenberg et al., 2017). This sex-specific finding may be because females are more likely to be subjected to sexualization and objectification both by society and themselves and thus are more self-conscious about the calorie consequences of alcohol consumption (Eisenberg et al., 2017).

#### 3.2.2 Weight status

Three studies reported potential relationships between weight-control compensatory behaviors and body mass index (BMI) (Eneva et al., 2017; Sob et al., 2021; Wammes et al., 2007). Compared to those with normal weight BMI, those with overweight or obesity had higher levels of compensatory behavior engagement (Wammes et al., 2007). A sex-stratified analysis revealed this association was only detected among males (Sob et al., 2021; Wammes et al., 2007)_._ Another study did not find significant associations between BMI and compensatory behaviors (Eneva et al., 2017). Moreover, whether compensatory behavior engagement predicts BMI over time is unclear; only one study assessed this longitudinally across two-years and found no relationship (Sob et al., 2021). There was also no significant association of BMI with alcohol consumption patterns or drunkorexia (Burke et al., 2010; Eisenberg et al., 2017; Laghi et al., 2019).

### 3.3 Types of weight-focused compensatory behaviors

#### 3.3.1 Eating behaviors

Eight articles focused on eating-related compensatory behaviors, although the measurement timescale (e.g., past week, past month, past year) and specific eating compensatory behaviors varied across the studies. The eating-related compensatory behaviors that were explored aligned with disordered eating behaviors (i.e., fasting, vomiting, and use of laxatives) and ranged from 11% in the past three months to 20% in the past year, to 24–38% lifetime prevalence (Anderson and Bulik, 2004; Bankoff et al., 2013; Dunn et al., 2003). Notably, the prevalence of fasting or non-purging related compensatory behaviors ranged from 68% in the past three months, to 29–53% lifetime prevalence (Anderson and Bulik, 2004; Dunn et al., 2003). One study explored the temporality of eating-related compensatory behaviors (Wammes et al., 2007). In this study, 32% of the total study sample reported eating less to compensate for overeating at least once a week (Wammes et al., 2007). Among the compensators, eating less was more prevalent the same day (14.1%) and on the day after (13.4%) of the overeating occasion, rather than the day before (2.5%) or within a few days after (12.0%) (Wammes et al., 2007). Most commonly, the compensators ate less between meals (29.3%) or ate less at dinner (12.7%) rather than with breakfast or lunch (<10%) (Wammes et al., 2007). Approximately one-fifth of the respondents reported utilizing multiple compensatory behaviors (such as restricting food and increasing physical activity) (Wammes et al., 2007). Only one study assessed compensation through altering food patterns or food choices (such as increased consumption of fruits and vegetables, or reduced consumption of sugar and fat) (Sob et al., 2021).

#### 3.3.2 Physical activity

Three articles directly examined the relationship between physical activity and compensatory behaviors and reported that the prevalence of exercise-related compensatory behaviors ranged from 7.9% to 59.5% (Anderson and Bulik, 2004; Blackstone and Herrmann, 2020; Eneva et al., 2017). The prevalence of exercising (36.2%) was higher than food-related compensatory behaviors such as restrictive eating (15.8%), vomiting (33.9%), and laxative use (14.1%) (Eneva et al., 2017). The intention to be physically active as a compensatory behavior was more strongly associated with one’s overall health status (Adkins and Keel, 2005). However, using exercise as a weight-focused compensatory behavior was associated with maladaptive behaviors and negative mental health indicators such as disordered eating, body dissatisfaction, internalization of the thin ideal, and psychological distress (Blackstone and Herrmann, 2020). Therefore, such a high prevalence for exercise engagement warrants research on its consequences.

Fitness wearables, a popular progress monitoring technology, may contribute to the engagement of weight-focused compensatory behaviors. One study found that when fitness goals set by the wearables were not met, nearly 70% of participants would engage in at least one compensatory behavior (e.g., eating less, increasing physical activity, delaying going to sleep to meet goals, exercising more vigorously) to meet the activity goal and 50% would engage in at least one compensatory behavior to meet a caloric goal (Blackstone and Herrmann, 2020). Females who engaged in exercise as a compensatory behavior were also more likely to demonstrate greater dietary restraint and had higher appearance dissatisfaction than those who did not use exercise as a compensatory behavior (Buchholz and Crowther, 2014). This is consistent with the general finding of other studies whereby exercise as a compensatory behavior was associated with worse eating, drinking, and exercising behaviors and poor self-esteem (e.g., disordered eating behaviors, exercise dependence, higher binge drinking frequency) (Buchholz and Crowther, 2014; Gorrell et al., 2019; Griffin and Vogt, 2021; Laghi et al., 2019). However, it is unclear if and what other weight-focused compensatory behaviors were used concurrently in many of these studies.

#### 3.3.3 Alcohol consumption

Twenty-one cross sectional studies highlighted drunkorexia (Bryant et al., 2012; Buchholz and Crowther, 2014; Burke et al., 2010; Castañeda et al., 2020; Choquette et al., 2018; Eisenberg and Fitz, 2014; Giles et al., 2009; Gorrell et al., 2019; Griffin and Vogt, 2021; Hill and Lego, 2019; Horvath et al., 2020; Hunt and Forbush, 2016; Knight et al., 2017; Laghi et al., 2019; Lupi et al., 2014; Martin et al., 2016; Michael and Witte, 2021; Moeck and Thomas, 2021; Peralta and Barr, 2016; Pompili and Laghi, 2018; Roosen and Mills, 2015). Within the population studied, drunkorexia prevalence ranged from 14.2 to 57.7% depending on the temporality (engagement of compensatory behaviors before, during, and after drinking) (Burke et al., 2010; Knight et al., 2017). In one study, 37.5% of participants skipped meals before drinking occasions, 46.3% consumed low-calorie or sugar-free alcoholic beverages during drinking, and 51.2% exercised after a drinking event for weight-control purpose (Knight et al., 2017).

Most commonly, individuals engaged in drunkorexia to compensate for the consequences of binge drinking (*n* = 7) (Bryant et al., 2012; Buchholz and Crowther, 2014; Burke et al., 2010; Castañeda et al., 2020; Knight et al., 2017; Lupi et al., 2014; Pompili and Laghi, 2018). Higher alcohol consumption was consistently associated with higher drunkorexia; that is the engagement and increased frequency of weight-control compensatory behaviors in response to alcohol (i.e., calorie restrictions, exercising) (Bryant et al., 2012; Buchholz and Crowther, 2014; Burke et al., 2010; Pompili and Laghi, 2018). Engagement of drunkorexia was also associated with higher intoxication rates and more binge drinking behavior (Giles et al., 2009; Knight et al., 2017; Moeck and Thomas, 2021). Among students, males who reported alcohol consumption-related compensatory behaviors were 1.99 times more likely to get drunk and females were 2.37 times more likely to get drunk in a typical week, respectively, than those of the same sex who do not engage in drunkorexia (Giles et al., 2009).

The most commonly employed weight-control compensatory behavior before drinking was to restrict food intake (e.g., skipping meals; 37.5%), during drinking was to consume low-calorie or sugar-free alcoholic beverages (46.3%), and after drinking was to exercise (51.2%) (Knight et al., 2017). Individuals also used purging methods (e.g., vomiting, using laxatives/diuretics) to compensate for alcohol consumption (Knight et al., 2017). Those who engaged in eating less or skipping meals with drinking were also significantly more likely to have motivations related to avoiding weight gain, more disordered eating behaviors (i.e., higher restraint and Eating Disorder Examination-Questionnaire scores), and poorer mental health (i.e., higher anxiety and depression scores) than individuals who report choosing to eat more food prior to drinking (Roosen and Mills, 2015).

Regional differences in drunkorexia engagement were also detected in a cross-country study conducted in the US and France (Choquette et al., 2018). While both French and American females engaged in comparable levels of drunkorexia (56.7 and 55.8% respectively), drive for thinness (i.e., one’s desire to be thin) and nationality significantly moderated the relationship between drinking and drunkorexia. At lower levels of drive for thinness, American females engaged in more drunkorexia than their French counterparts but at higher levels of drive for thinness, French participants were more likely to engage in drunkorexia for compensatory purposes than Americans. This interaction and corresponding cross-cultural differences in this study warrant an in-depth look into ethnicity since different ethnicities have diverse cultures.

### 3.4 Correlates and predictors

#### 3.4.1 General compensatory behaviors

The majority of the studies assessing psychological well-being reported significant relationships between weight-control compensatory behaviors and worse psychological well-being (Bankoff et al., 2013; Castañeda et al., 2020; Horvath et al., 2020; Laghi et al., 2019; LePage et al., 2008; Roosen and Mills, 2015). For instance, poor psychosocial functioning (the ability to manage one’s own mental well-being and social relationships) and greater perceived vulnerability to disease were associated with weight-control compensatory behaviors (Bankoff et al., 2013; Hoover et al., 2023). Three cross-sectional studies explored whether an individual’s ability to identify different emotions (e.g., distinguishing between feeling angry and guilty) was associated with their compensatory behaviors (Castañeda et al., 2020; Horvath et al., 2020; Laghi et al., 2019). All three studies found that lower ability to differentiate between emotions was associated with higher weight-focused compensatory behaviors engagement (Castañeda et al., 2020; Horvath et al., 2020; Roosen and Mills, 2015). However, how much emotional differentiation was associated with compensatory behaviors varied among these studies. Overall, Castañeda et al. (2020) found a significant association only between poor negative emotion differentiation abilities (e.g., not being able to distinguish between guilt and anger) and only with increased compensatory behaviors frequency. The authors surmised that individuals with difficulties differentiating between negative emotions may use compensatory behaviors to try to broadly alleviate negative emotions by gaining a sense of control because they are unable to find better adaptive methods to manage their emotions.

Moreover, quality of interpersonal relationships such as relationship avoidance (fear of intimacy), or history of experiencing intimate partner violence emerged as unique correlates for general compensatory behaviors (Bankoff et al., 2013; Holmes et al., 2023). Both of these correlates may negatively influence one’s eating behavior and overall psychological functioning, and thus correlate with engagement in weight-control compensatory behaviors (O’Shaughnessy and Dallos, 2009). Body esteem, internalization of a thin ideal, and internalized weight bias were strongly associated with higher compensatory behaviors frequency and endorsing more types of compensatory behaviors simultaneously (Hoover et al., 2023; LePage et al., 2008; McCabe et al., 2023).

While there were significant negative associations between compensatory behaviors and psychological wellbeing, the relationships between compensatory behaviors, health behaviors and physical health were less clear. Only one study found that compensatory behaviors significantly improved diet quality and increased physical activity levels over two years (Sob et al., 2021). However, this positive finding had a small effect size where compensatory behaviors explained less than 1% of the variance in diet quality and physical activity change over time. The study also had a 50% drop out rate and thus the results might be confounded with attrition bias.

#### 3.4.2 Drunkorexia

Twenty-one articles examined the associations of drunkorexia with disordered eating and substance/alcohol use (Bryant et al., 2012; Buchholz and Crowther, 2014; Burke et al., 2010; Castañeda et al., 2020; Choquette et al., 2018; Eisenberg et al., 2017; Eisenberg and Fitz, 2014; Giles et al., 2009; Gorrell et al., 2019; Griffin and Vogt, 2021; Hill and Lego, 2019; Horvath et al., 2020; Hunt and Forbush, 2016; Knight et al., 2017; Laghi et al., 2019; Lupi et al., 2014; Martin et al., 2016; Michael and Witte, 2021; Peralta and Barr, 2016; Pompili and Laghi, 2018; Roosen and Mills, 2015). Findings showed drunkorexia to be more related to disordered eating than alcohol use, but only in females (Gorrell et al., 2019; Hunt and Forbush, 2016). Preliminary evidence suggests that these sex-specific associations may be confounded by different personality qualities, namely body esteem and whether the person was inclined to pursue novel experiences (“sensation seeking”). Greater binge drinking frequency and eating disorder symptomatology also made an independent and significant association with drunkorexia behaviors (Knight et al., 2017). Laghi et al. (2019) found poorer emotional differentiation abilities as a positive correlate of drunkorexia. Indeed, Horvath et al. (2020) suggested that emotion dysregulation may be indirectly related to drunkorexia by affecting disordered eating and alcohol use because drunkorexia was no longer significant after accounting for disordered eating, alcohol use and problems, and BMI from regression models. These studies suggested that deficits in emotion management may contribute to the engagement of general and alcohol-related weight-control compensatory behaviors.

Other potential correlates of drunkorexia included: higher hazardous drinking level (incorporates alcohol consumption frequency, quantity, and binge drinking) and stronger weight or shape control motivations (Martin et al., 2016; Michael and Witte, 2021). Additionally, older adolescents/young adults with high asceticism (i.e., denial of desires and abstinence from indulgence) might employ drunkorexia as a behavior to gain a sense of independence and control (Laghi et al., 2019).

## 4 Discussion

This review paper examined the literature on weight-control compensatory behaviors, in particular, the patterns and correlates of such behaviors with health, including eating and exercising behaviors, as well as psychological well-being. Our review found different engagement patterns for weight-control compensatory behaviors across biological sex and weight status. Although both males and females engaged in compensatory behaviors, females had a higher frequency of engagement. This reflected females’ greater desire to control their weight, potentially resulting from females having higher levels of body shape concerns than males due to the societal objectification of female bodies (Eisenberg et al., 2017; Eisenberg and Fitz, 2014). Males and females also used different compensatory behaviors; males engaged in physical activity while females changed their eating patterns as a compensatory strategy (Anderson and Bulik, 2004).

The study findings suggest that weight-control compensatory behaviors were generally associated with negative mental health and psychological measures such as greater body dissatisfaction, higher internalization of the thin ideal, and greater psychological distress (Bankoff et al., 2013; Castañeda et al., 2020; Horvath et al., 2020; Laghi et al., 2019; LePage et al., 2008; Roosen and Mills, 2015). One mediating factor of such negative association is that weight-control compensatory behaviors may not result in achieving weight management goals. Although someone with a weight control motive might exercise more to counteract their calorie intake, people tend to underestimate calories in food, and overestimate energy expenditure from exercise (Block et al., 2013; Werle et al., 2011). As a result, a caloric surplus may still occur. Findings from controlled lab studies support this; although there is a lot of variability and individual motivations for compensatory behaviors, full caloric compensation is not typical (Finlayson et al., 2009; Hopkins et al., 2014). These negative psychological consequences indicate a possibility that the compensation mindset and poor mental health may reinforce each other, especially when individuals fail to achieve their weight management goals using compensatory behaviors.

The relationship between weight-focused compensatory behaviors and physical health was less clear. Studies have primarily focused on examining sex differences in weight-focused compensatory behaviors, with less attention to weight-status or physical health (Amrein et al., 2017; Eneva et al., 2017; Sob et al., 2021). For weight status, the associations between BMI and weight-control compensatory behaviors were mixed, possibly due to the limitations of BMI as an imperfect proxy for adiposity. Further research in the links between compensatory behaviors and physical health is needed.

Compensatory physical activity is another area of focus that is important to further investigate in diverse populations since individuals may perceive exercising as more socially acceptable than the other forms of compensatory behaviors (Buchholz and Crowther, 2014). Studies in this review found that performing physical activity with a weight-control compensatory mindset were associated with unhealthy lifestyle behaviors around eating, drinking, and exercising as well as poor self-esteem. However, another review determined that exercise resulted in a decrease of non-exercise physical activities in the majority of studies (Mansfeldt and Magkos, 2023). Thus, these reviews suggests that the underlying intention to be physically active as a weight-control compensatory behavior in tandem with other weight control compensatory behaviors is an important caveat to the literature and needs to be further explored.

Finally, this paper presents findings on drunkorexia, a special area of weight related compensatory behavior. Results were consistent with the prevalence estimates, and correlates of drunkorexia noted in a recently published systematic review (Berry et al., 2024). This scoping review extends that literature to other compensatory behaviors. Nevertheless, a major limitation within the body of research is the limited sample age diversity. Future research should consider examining different populations and age ranges. Moreover, drunkorexia’s association with binge drinking warrants an investigation on the direction of causation to inform relevant health promotion measures.

### 4.1 Strength and limitations

Our review extends the literature and has noticeable strengths. Multiple database platforms and reference lists were assessed by multiple independent reviewers. To our knowledge, this is the first study to review weight-focused compensatory behaviors. The findings of this review may be valuable for identifying gaps within the literature to provide directions for future research and provide insight into developing health promotion strategies addressing weight-focused compensatory behaviors. Despite these strengths, this study is not without limitations. Many of these studies assessed in this review were of low quality, suggesting there is a need to create a standardized measure, as well as more rigorous sampling methodology such as using random, large, and/or representative samples with diverse backgrounds, ensuring internal consistency, as well as reporting on response rate and addressing missing data. All except one study (Giles et al., 2009) used convenience samples, and the only study (Giles et al., 2009) that utilized random sampling chose one university as their sampling frame. The sample populations were also predominantly white female university students. Although several studies noted significant interaction terms, results were not stratified or interpreted given the interactions.

Another notable limitation is that the literature used sex and gender interchangeably while almost exclusively only conducting sex comparisons. Only one of the studies in this review specifically assessed gender differences (Martin et al., 2016). This review cannot distinguish between the two due to limited gender data. While sex differences between males and females may appear like gender differences between men and women, distinctions should be investigated in future studies. In addition, as gender minorities have reported disproportionately elevated risks for various health conditions including eating disorders and disordered appearance control behaviors, future studies should be inclusive of all gender identities (Calzo et al., 2017).

### 4.2 Direction for future research

Compiled study findings in this review suggested that compensatory behaviors are a commonly utilized method of weight management. Although greater use of compensatory behaviors was associated with worse psychological well-being, causality cannot be inferred due to the cross-sectional nature of most studies. The evidence between compensatory behaviors and physical health measures was less clear. The long-term mental and physical health impacts are also unknown. Relatedly, the types of compensatory behaviors commonly investigated were extreme behaviors (such as use of laxatives or vomiting). Studies in this review also focused on examining relationships between singular compensatory behaviors and health indicators. Future studies may investigate the engagement in multiple compensatory behaviors as individuals may utilize several compensatory behaviors at once to control their weight (i.e., restricting food and increasing exercise). Whether compensatory behaviors that are aligned with recommended behaviors such as national dietary and physical activity guidelines have not been adequately explored. Future research should utilize more sound methodologies to examine the above-mentioned correlates and establish the direction of causation.

Considering the diversity among demographic characteristics, potential patterns for future examination include age, ethnicity, gender identity, sexual orientation, and socioeconomic characteristics. These factors are strongly associated with weight-control intentions, one’s health status, and the quality of healthcare they receive (Braveman and Gottlieb, 2014; Fiscella and Sanders, 2016; Higashi et al., 2004; Luker et al., 2011).

## 5 Conclusion

It is important to distinguish compensatory behaviors from health behaviors as certain compensatory behaviors can be detrimental to health. Differences in weight-focused compensatory behavior patterns were found across sex and weight status. Notable correlates for both general compensatory behaviors and drunkorexia include low body esteem, internalization of a thin ideal, and poor emotion management. Unique to general compensatory behaviors, quality of interpersonal relationships, experiences of intimate partner violence, and greater perceived vulnerability of disease emerged as correlates. Specifically, for drunkorexia, sensation seeking tendencies, binge drinking frequency, and eating disorder symptomatology are major correlates.

Psychological well-being and specific psychological factors emerged as significantly associated with weight-focused compensatory behaviors. Additionally, weight-focused compensatory behaviors may have consequences including changes in diet quality, physical activity level, and alcohol consumptions patterns. However, since the above findings were based on a limited number of studies of moderate quality, more research that is methodologically rigorous is needed. In particular, the development of standardized measures and guidelines for weight-focused compensatory behaviors is imperative as it would allow a more comprehensive investigation of these behaviors and their role in individual and population health.

## Data Availability

The original contributions presented in the study are included in the article/supplementary material, further inquiries can be directed to the corresponding author.

## References

[ref1] Adkins E. C. Keel P. K. (2005). Does “excessive” or “compulsive” best describe exercise as a symptom of bulimia nervosa? Int. J. Eat. Disord. 38, 24–29. doi: 10.1002/eat.20140, PMID: 15991218

[ref2] Amrein M. A. Rackow P. Inauen J. Radtke T. Scholz U. (2017). The role of compensatory health beliefs in eating behavior change: a mixed method study. Appetite 116, 1–10. doi: 10.1016/j.appet.2017.04.016, PMID: 28433774

[ref3] Anderson C. B. Bulik C. M. (2004). Gender differences in compensatory behaviors, weight and shape salience, and drive for thinness. Eat. Behav. 5, 1–11. doi: 10.1016/j.eatbeh.2003.07.001, PMID: 15000949

[ref4] Bankoff S. M. Valentine S. E. Jackson M. A. Schacht R. L. Pantalone D. W. (2013). Compensatory weight control behaviors of women in emerging adulthood: associations between childhood abuse experiences and adult relationship avoidance. J Am Coll Health 61, 468–475. doi: 10.1080/07448481.2013.833515, PMID: 24152024

[ref5] Berry K. A. Choquette E. M. Looby A. Rancourt D. (2024). Unification of the food and alcohol disturbance literature: a systematic review. Clin. Psychol. Rev. 113:102486. doi: 10.1016/j.cpr.2024.102486, PMID: 39168054

[ref6] Blackstone S. R. Herrmann L. K. (2020). Fitness wearables and exercise dependence in college women: considerations for university health education specialists. Am. J. Health Educ. 51, 225–233. doi: 10.1080/19325037.2020.1767004

[ref7] Block J. P. Condon S. K. Kleinman K. Mullen J. Linakis S. Rifas-Shiman S. . (2013). Consumers’ estimation of calorie content at fast food restaurants: cross sectional observational study. BMJ 346:f2907. doi: 10.1136/bmj.f2907, PMID: 23704170 PMC3662831

[ref8] Braveman P. Gottlieb L. (2014). The social determinants of health: It’s time to consider the causes of the causes. Public Health Rep. 129, 19–31. doi: 10.1177/00333549141291S206, PMID: 24385661 PMC3863696

[ref9] Bryant J. B. Darkes J. Rahal C. (2012). College students’ compensatory eating and behaviors in response to alcohol consumption. J. Am. Coll. Heal. 60, 350–356. doi: 10.1080/07448481.2011.630702, PMID: 22686357

[ref10] Buchholz L. J. Crowther J. H. (2014). Women who use exercise as a compensatory behavior: how do they differ from those who do not? Psychol. Sport Exerc. 15, 668–674. doi: 10.1016/j.psychsport.2014.06.010

[ref11] Burke S. C. Cremeens J. Vail-Smith K. Woolsey C.. (2010). Drunkorexia: calorie restriction prior to alcohol consumption among college freshman. J. Alcohol Drug Educ. 54, 17–32.

[ref12] Calzo J. P. Blashill A. J. Brown T. A. Argenal R. L. (2017). Eating disorders and disordered weight and shape control behaviors in sexual minority populations. Curr. Psychiatry Rep. 19:49. doi: 10.1007/s11920-017-0801-y, PMID: 28660475 PMC5555626

[ref13] Castañeda G. Colby S. E. Barnett T. E. Olfert M. D. Zhou W. Leite W. L. . (2020). Examining the effect of weight conscious drinking on binge drinking frequency among college freshmen. J. Am. Coll. Heal. 68, 906–913. doi: 10.1080/07448481.2019.1642204, PMID: 31348733

[ref14] Choquette E. M. Ordaz D. L. Melioli T. Delage B. Chabrol H. Rodgers R. . (2018). Food and alcohol disturbance (FAD) in the U.S. and France: nationality and gender effects and relations to drive for thinness and alcohol use. Eat. Behav. 31, 113–119. doi: 10.1016/j.eatbeh.2018.09.002, PMID: 30245363

[ref15] Colleen Stiles-Shields E. Labuschagne Z. Goldschmidt A. B. Doyle A. C. Grange D. L. (2012). The use of multiple methods of compensatory behaviors as an indicator of eating disorder severity in treatment-seeking youth. Int. J. Eat. Disord. 45, 704–710. doi: 10.1002/eat.22004, PMID: 22331840 PMC3355214

[ref16] Diep C. S. Hingle M. Chen T.-A. Dadabhoy H. R. Beltran A. Baranowski J. . (2015). The automated self-administered 24-hour dietary recall for children, 2012 version, for youth aged 9 to 11 years: a validation study. J. Acad. Nutr. Diet. 115, 1591–1598. doi: 10.1016/j.jand.2015.02.021 25887784 PMC4584168

[ref17] Downes M. J. Brennan M. L. Williams H. C. Dean R. S. (2016). Development of a critical appraisal tool to assess the quality of cross-sectional studies (AXIS). BMJ Open 6:e011458. doi: 10.1136/bmjopen-2016-011458, PMID: 27932337 PMC5168618

[ref18] Dunn E. C. Neighbors C. Larimer M. (2003). Assessing readiness to change binge eating and compensatory behaviors. Eat. Behav. 4, 305–314. doi: 10.1016/S1471-0153(03)00023-0, PMID: 15000973

[ref19] Eisenberg M. H. Fitz C. C. (2014). “Drunkorexia”: exploring the who and why of a disturbing trend in college students’ eating and drinking behaviors. J. Am. Coll. Heal. 62, 570–577. doi: 10.1080/07448481.2014.947991, PMID: 25102366

[ref20] Eisenberg M. H. Johnson C. C. Zucker A. N. (2017). Starving for a drink: sexual objectification is associated with food-restricted alcohol consumption among college women, but not among men. Women Health 58, 175–187. doi: 10.1080/03630242.2017.1292342, PMID: 28277155

[ref21] Eneva K. T. Murray S. O’Garro-Moore J. Yiu A. Alloy L. B. Avena N. M. . (2017). Reward and punishment sensitivity and disordered eating behaviors in men and women. J. Eat. Disord. 5:6. doi: 10.1186/s40337-017-0138-2, PMID: 28228946 PMC5311722

[ref22] Fairburn C. G. Beglin S. J. (1994). Assessment of eating disorders: interview or self-report questionnaire? Int. J. Eat. Disord. 16, 363–370. doi: 10.1002/1098-108X(199412)16:4<363::AID-EAT2260160405>3.0.CO;2-# 7866415

[ref23] Finlayson G. Bryant E. Blundell J. E. King N. A. (2009). Acute compensatory eating following exercise is associated with implicit hedonic wanting for food. Physiol. Behav. 97, 62–67. doi: 10.1016/j.physbeh.2009.02.002, PMID: 19419671

[ref24] Fiscella K. Sanders M. R. (2016). Racial and ethnic disparities in the quality of health care. Annu. Rev. Public Health 37, 375–394. doi: 10.1146/annurev-publhealth-032315-021439 26789384

[ref25] Forestier C. Sarrazin P. Sniehotta F. Allenet B. Heuzé J. P. Gauchet A. . (2020). Do compensatory health beliefs predict behavioural intention in a multiple health behaviour change context? Evidence in individuals with cardiovascular diseases? Psychol. Health Med. 25, 593–600. doi: 10.1080/13548506.2019.1653476 31402693

[ref26] Fuller-Tyszkiewicz M. Rodgers R. F. Maïano C. Mellor D. Sicilia A. Markey C. H. . (2022). Testing of a model for risk factors for eating disorders and higher weight among emerging adults: baseline evaluation. Body Image 40, 322–339. doi: 10.1016/j.bodyim.2022.01.007 35121568

[ref27] Giles S. M. Champion H. Sutfin E. L. McCoy T. P. Wagoner K. (2009). Calorie restriction on drinking days: an examination of drinking consequences among college students. J. Am. Coll. Heal. 57, 603–610. doi: 10.3200/JACH.57.6.603-610, PMID: 19433398 PMC3710706

[ref28] Gorrell S. Walker D. C. Anderson D. A. Boswell J. F. (2019). Gender differences in relations between alcohol-related compensatory behavior and eating pathology. Eat. Weight Disord. 24, 715–721. doi: 10.1007/s40519-018-0545-7 30196525

[ref29] Griffin B. L. Vogt K. S. (2021). Drunkorexia: is it really “just” a university lifestyle choice? Eat. Weight Disord. 26, 2021–2031. doi: 10.1007/s40519-020-01051-x 33125626 PMC8292268

[ref30] Higashi T. Shekelle P. Solomon D. Knight E. L. Roth C. P. Chang J. T. . (2004) Quality of health care received by older adults. RAND Corporation. Available at: https://www.rand.org/pubs/research_briefs/RB9051.html (accessed 18 December 2021).

[ref31] Hill E. M. Lego J. E. (2019). Examining the role of body esteem and sensation seeking in drunkorexia behaviors. Eat. Weight Disord. 25, 1507–1513. doi: 10.1007/s40519-019-00784-8 31587177

[ref32] Holmes S. C. King K. C. Gonzalez A. Norton M. K. Silver K. E. Sullivan T. P. . (2023). Associations among intimate partner violence, posttraumatic stress disorder symptoms, and disordered eating among women intimate partner violence survivors residing in shelter. J. Interpers. Violence 38, NP2135–NP2158. doi: 10.1177/08862605221098968 35536767 PMC9993353

[ref33] Hoover L. V. Ackerman J. M. Cummings J. R. Gearhardt A. N. (2023). The Association of Perceived Vulnerability to disease with cognitive restraint and compensatory behaviors. Nutrients 15:8. doi: 10.3390/nu15010008 PMC9824184 36615665

[ref34] Hopkins M. Blundell J. E. King N. A. (2014). Individual variability in compensatory eating following acute exercise in overweight and obese women. Br. J. Sports Med. 48, 1472–1476. doi: 10.1136/bjsports-2012-091721, PMID: 23666018

[ref35] Horvath S. A. Shorey R. C. Racine S. E. (2020). Emotion dysregulation as a correlate of food and alcohol disturbance in undergraduate students. Eat. Behav. 38:101409. doi: 10.1016/j.eatbeh.2020.101409 32585563

[ref36] Hunt T. K. Forbush K. T. (2016). Is “drunkorexia” an eating disorder, substance use disorder, or both? Eat. Behav. 22, 40–45. doi: 10.1016/j.eatbeh.2016.03.034, PMID: 27085168

[ref37] Knäuper B. Rabiau M. (2004). Compensatory health beliefs: scale development and psychometric properties. Psychol. Health 19, 607–624. doi: 10.1080/088704404200019673

[ref38] Knight A. Castelnuovo G. Pietrabissa G. Manzoni G. M. Simpson S. (2017). Drunkorexia: an empirical investigation among Australian female university students. Aust. Psychol. 52, 414–423.

[ref39] Laghi F. Pompili S. Bianchi D. Lonigro A. Baiocco R. (2019). Psychological characteristics and eating attitudes in adolescents with drunkorexia behavior: an exploratory study. Eat. Weight Disord. 25, 709–718. doi: 10.1007/s40519-019-00675-y, PMID: 30888609

[ref40] LePage M. L. Crowther J. H. Harrington E. F. Engler P. (2008). Psychological correlates of fasting and vigorous exercise as compensatory strategies in undergraduate women. Eat. Behav. 9, 423–429. doi: 10.1016/j.eatbeh.2008.06.002, PMID: 18928905

[ref41] Luker J. A. Wall K. Bernhardt J. Edwards I. Grimmer-Somers K. A. (2011). Patients’ age as a determinant of care received following acute stroke: a systematic review. BMC Health Serv. Res. 11:161. doi: 10.1186/1472-6963-11-161, PMID: 21729329 PMC3150246

[ref42] Lupi M. Acciavatti T. Santacroce R. Cinosi E. (2014). Drunkorexia: a pilot study in an Italian sample. Res. Adv. Psychiatry 1, 1–5.

[ref43] Mansfeldt J. M. Magkos F. (2023). Compensatory responses to exercise training as barriers to weight loss: changes in energy intake and non-exercise physical activity. Curr. Nutr. Rep. 12, 327–337. doi: 10.1007/s13668-023-00467-y, PMID: 36933180

[ref44] Martin R. J. Chaney B. H. Vail-Smith K. Gallucci A. R. (2016). Hazardous drinking and weight-conscious drinking behaviors in a sample of college students and college student athletes. Subst. Abus. 37, 488–493. doi: 10.1080/08897077.2016.1142922, PMID: 26820817

[ref45] Mauldin K. May M. Clifford D. (2022). The consequences of a weight-centric approach to healthcare: a case for a paradigm shift in how clinicians address body weight. Nutr. Clin. Pract. 37, 1291–1306. doi: 10.1002/ncp.10885 35819360

[ref46] McCabe M. Alcaraz-Ibanez M. Markey C. Sicilia A. Rodgers R. F. Aimé A. . (2023). A longitudinal evaluation of a biopsychosocial model predicting BMI and disordered eating among young adults. Aust. Psychol. 58, 57–79. doi: 10.1080/00050067.2023.2181686

[ref47] Michael M. L. Witte T. H. (2021). Traumatic stress and alcohol-related disordered eating in a college sample. J. Am. Coll. Heal. 69, 806–811. doi: 10.1080/07448481.2019.1706534 31944908

[ref48] Moeck E. K. Thomas N. A. (2021). Food and alcohol disturbance in a broad age-range adult sample. Eat. Behav. 41:101510. doi: 10.1016/j.eatbeh.2021.101510 33901799

[ref49] Mond J. M. Hay P. J. Rodgers B. Owen C. Beumont P. J. V. (2004). Validity of the eating disorder examination questionnaire (EDE-Q) in screening for eating disorders in community samples. Behav. Res. Ther. 42, 551–567. doi: 10.1016/S0005-7967(03)00161-X, PMID: 15033501

[ref50] O’Shaughnessy R. Dallos R. (2009). Attachment research and eating disorders: a review of the literature. Clin. Child Psychol. Psychiatry 14, 559–574. doi: 10.1177/1359104509339082, PMID: 19759074

[ref51] Peralta R. L. Barr P. B. (2016). Gender orientation and alcohol-related weight control behavior among male and female college students. J. Am. Coll. Heal. 65, 229–242. doi: 10.1080/07448481.2016.1271802, PMID: 27982766

[ref52] Pompili S. Laghi F. (2018). Drunkorexia: disordered eating behaviors and risky alcohol consumption among adolescents. J. Health Psychol. 25, 2222–2232. doi: 10.1177/1359105318791229, PMID: 30073869

[ref53] Radtke T. Kaklamanou D. Scholz U. Hornung R. Armitage C. J. (2014). Are diet-specific compensatory health beliefs predictive of dieting intentions and behaviour? Appetite 76, 36–43. doi: 10.1016/j.appet.2014.01.014 24472827

[ref54] Rahal C. J. Bryant J. B. Darkes J. Menzel J. E. Thompson J. K. (2012). Development and validation of the compensatory eating and behaviors in response to alcohol consumption scale (CEBRACS). Eat. Behav. 13, 83–87. doi: 10.1016/j.eatbeh.2011.11.001, PMID: 22365787

[ref55] Roosen K. M. Mills J. S. (2015). Exploring the motives and mental health correlates of intentional food restriction prior to alcohol use in university students. J. Health Psychol. 20, 875–886. doi: 10.1177/1359105315573436, PMID: 26032803

[ref56] Sob C. Siegrist M. Hagmann D. Hartmann C. (2021). A longitudinal study examining the influence of diet-related compensatory behavior on healthy weight management. Appetite 156:104975. doi: 10.1016/j.appet.2020.104975, PMID: 32966848

[ref57] Staples C. Rancourt D. (2022). Testing the interaction of thinness/restriction and negative affect reduction expectancies on disordered eating behavior. Eat. Behav. 47:101663. doi: 10.1016/j.eatbeh.2022.101663 36067649

[ref58] Stice E. Telch C. F. Rizvi S. L. (2000). Development and validation of the eating disorder diagnostic scale: a brief self-report measure of anorexia, bulimia, and binge-eating disorder. Psychol. Assess. 12, 123–131. doi: 10.1037/1040-3590.12.2.123 10887758

[ref59] Tylka T. L. Annunziato R. A. Burgard D. Daníelsdóttir S. Shuman E. Davis C. . (2014). The weight-inclusive versus weight-normative approach to health: evaluating the evidence for prioritizing well-being over weight loss. J. Obes. 2014:e983495, 1–18. doi: 10.1155/2014/983495 PMC4132299 25147734

[ref60] Wammes B. French S. Brug J. (2007). What young Dutch adults say they do to keep from gaining weight: self-reported prevalence of overeating, compensatory behaviours and specific weight control behaviours. Public Health Nutr. 10, 790–798. doi: 10.1017/S1368980007258537 17381910

[ref61] Werle C. O. C. Wansink B. Payne C. R. (2011). Just thinking about exercise makes me serve more food. Physical activity and calorie compensation. Appetite 56, 332–335. doi: 10.1016/j.appet.2010.12.016, PMID: 21185895

[ref62] Williams-Kerver G. A. Crowther J. H. (2020). Emotion differentiation and disordered eating behaviors: the role of appearance schemas. Eat. Behav. 37:101369. doi: 10.1016/j.eatbeh.2020.101369, PMID: 32087556 PMC7246154

[ref63] World Health Organization (1948). Constitution of the World Health Organization. Available at: https://apps.who.int/gb/bd/PDF/bd47/EN/constitution-en.pdf?ua=1 (Accessed September 20, 2024).

[ref64] Zhao K. Xu X. Zhu H. Xu Q. (2021). Compensatory belief in health behavior management: a concept analysis. Front. Psychol. 12:3765. doi: 10.3389/fpsyg.2021.705991 PMC8429599 34512462

